# Electroacupuncture reduces scopolamine-induced amnesia via mediating the miR-210/SIN3A and miR-183/SIN3A signaling pathway

**DOI:** 10.1186/s10020-020-00233-8

**Published:** 2020-11-12

**Authors:** Fan Ye, Shiming Tian, Huimin Hu, Zhengwen Yu

**Affiliations:** Department of Anesthesiology, Xiangyang Central Hospital, Affiliated Hospital of Hubei University of Arts and Science, No.136, Jingzhou Street, Xiangcheng District, Xiangyang, 441021 Hubei China

**Keywords:** Electroacupuncture, Scopolamine, SIN3A, miR-210, miR-183, Cognitive impairment

## Abstract

**Background:**

The expression of SIN3A is closely correlated with electroacupuncture (EA) treatment efficacy of scopolamine-induced amnesia (SIA), but its underlying mechanisms remain to be further explored.

**Methods:**

Quantitative real-time PCR was performed to analyze the expression of candidate microRNAs (miRNAs) and SIN3A mRNA in a rat model of SIA. Western blot was carried out to evaluate the differential expression of SIN3A proteins under different circumstances. Luciferase assay was used to explore the inhibitory role of certain miRNAs in SIN3A expression. A novel object recognition (NOR) test was performed to assess the memory function of SIA rats undergoing EA treatment. Immunohistochemistry was carried out to evaluate the expression of SIN3A in the hippocampus of SIA rats.

**Results:**

Rno-miR-183-5p, rno-miR-34c-3p and rno-miR-210-3p were significantly up-regulated in SIA rats treated with EA. In addition, rno-miR-183-5p and rno-miR-210-3p exerted an inhibitory effect on SIN3A expression. EA treatment of SIA rats effectively restored the dysregulated expression of rno-miR-183-5p, rno-miR-210-3p and SIN3A. EA treatment also promoted the inhibited expression of neuronal IEGs including Arc, Egr1, Homer1 and Narp in the hippocampus of SIA rats. Accordingly, the NOR test also confirmed the effect of EA treatment on the improvement of memory in SIA rats.

**Conclusion:**

In summary, the findings of this study demonstrated that scopolamine-induced amnesia was associated with downregulated expression of miR-210/miR-183 and upregulated expression of SIN3A. Furthermore, treatment with EA alleviated scopolamine-induced amnesia in rats and was associated with upregulated expression of miR-210/miR-183 and downregulated expression of SIN3A.

## Introduction

Scopolamine is a strong amnestic compound with the ability to block the activity of muscarinic acetylcholine receptors to impair the memory and learning functions in mammals (Wang et al. 2010). Various amnesia models established by induction using scopolamine have been commonly used to screen potential drugs with the ability to enhance memory (Eun et al. 2017). Moreover, other applications of scopolamine were also reported. For example, scopolamine is also an anticholinergic agent used for the prevention of motion sickness-associated nausea and vomiting (Ebert and Kirch 1998). And scopolamine was also demonstrated to selectively enhance the generalization of odor representations in the rat olfactory cortex (Wilson 2001).

Electroacupuncture (EA) was initially used in European countries such as Italy and France in the nineteenth century (Xiang et al. 2017). At this moment, EA became a popular type of traditional Chinese medicine in the treatment of a wide range of medical conditions (Wang and Wu 2017). The electro stimulations of anatomical positions generated by EA can affect various physiological processes, such as angiogenesis, tissue regeneration and neuron activation, which may be promoted by the increased levels of expression of some genes in the nervous system induced by EA (Pomeranz 1996; Melzack and Wall 1965).

MicroRNAs belong to a class of short non-coding RNA transcripts that can modulate the expression of target genes by either translational suppression or mRNA degradation. Since miRNAs can sustain repeated freeze–thaw cycles, they are superior to protein biomarkers in the diagnosis of many medical disorders. For example, one miRNA, miRNA-210, can increase the expression of vascular endothelial growth factor (VEGF) to enhance the proliferation of vascular cells (Alaiti et al. 2012; Zeng et al. 2014; Lou et al. 2012; Shoji et al. 2012).

Highly expressed in non-neuronal as well as neuronal cells, SIN3A contains different domains, such as a paired helix domain, several core elements, a domain for histone deacetylase (HDAC) interaction, as well as a highly conserved domain. Via its domain for HDAC interaction, SIN3A can bind to a RBAP46/48 protein core complex for histone binding, a SAP18/20 protein for complex stabilization, as well as an HDAC2 enzyme (Grzenda et al. 2009). The silencing of SIN3A in rats can rescue the impaired memory functions caused by the exposure to scopolamine. Furthermore, the silencing of SIN3A in the hippocampus of rats with amnesia elevated the levels of H3K9 and H3K14 acetylation in the promoter of immediate early genes (IEGs), while suppressing the binding affinity of SIN3A for the promoter of IEGs in neuron cells, thus restoring the memory functions in these rats (Srivas and Thakur 2018). Neuronal IEGs are a family of synaptic plasticity genes involved in memory consolidation (Abel and Lattal 2001a). And regulatory transcription factors such as Egr1 and effector proteins such as Arc, Homer1 and Narp are encoded by IEGs. Especially, Egr1 can control the expression of synaptic plasticity genes, Arc can control actin dynamics, Homer1 is involved in glutamate receptor transportation, and Narp can regulate glutamate receptor clustering at the synapse (Loebrich and Nedivi 2009).

It has been reported that EA treatment continuously increased the expression of several candidate miRNAs including rno-miR-183-5p, rno-miR-34c-3p, rno-miR-210-3p, rno-miR-758-5p, rno-miR-568, and rno-miR-196c-3p (Zheng et al. 2016). Meanwhile, SIN3A silencing was found to mitigate SIA (Srivas and Thakur 2018). In this study, we set up an animal model of SIA and treated it with or without EA to investigate the effect of EA on SIA.

## Materials and methods

### Animal grouping and treatment

In this study, 25 Wistar male rats were acquired from the Center of Laboratory Animals at our institute. All rats were about 8 weeks in age and weighed 250 to 300 g. The animal protocols of this study have been approved by the Institutional Animal Ethics Committee. After 7 days of adaptation, 24 rats were randomly selected and divided into 4 groups with 6 rats in each group, i.e., 1. SHAM group; 2. SIA group; 3. SIA + EA group; and 4. EA group. Comparisons were made among the SHAM group, SIA group and SIA + EA group to study the effect of EA on SIA rats. And comparisons were also made between the SHAM group and EA group to identify potential miRNAs that responded to EA treatment. All rats were placed in individual cages at 22 ± 1 °C during the experiment. The establishment of the SIA model and corresponding EA treatment will be described below.

### Establishment of an SIA rat model

Scopolamine hydrobromide (Sigma-Aldrich, St. Louis, MO) was dissolved in 0.9% saline and then injected into rats via intraperitoneal injection at a dosage of 3 mg/kg. The injection was carried out once a day for seven days consecutively. The rats in the SHAM group were given the injection of 0.9% saline at the same dosage and the same frequency. After the 7 days of consecutive injections were finished, all rats were euthanized by CO_2_ suffocation to collect their brain tissues for subsequent analyses.

### EA treatment

The treatment with EA was carried out immediately after the SIA rats gained full consciousness after the induction of anesthesia. According to previous publications (Chang et al. 2018), the acupuncture site of Renzhong located at the junction beneath the nasal septum along the cleft lip midline, as well as the Neiguan site located at the junction between the flexor carpi radialis and palmaris longus, were subjected to electrostimulation delivered via a 0.25 mm × 30 mm single-use needle. During the EA procedure, the frequency of electro stimulation was set to 2 Hz, while the intensity of the electro stimulation was set to 3.0 mA. The EA stimulation was applied for 1 min in 12 h intervals on the rats immediately after full recovery from anesthesia until the rats were sacrificed.

### Measurement of rat model memory consolidation

The Novel object recognition (NOR) test was carried out to assess recognition memory according to a procedure described elsewhere (Singh and Thakur 2014). In brief, we used objects of similar sizes with different shapes in the experiment. For the habituation process, each rat was placed in the box for 5 min on two successive days before returning to the home cage. On the third day, two similar objects were placed and rats were allowed to interact with the objects for 5 min and before returning to the home cage. After 48 h of training, we replaced one object with a novel object before re-introducing the rats to interact with the objects. The objects and box were wiped by 70% ethanol after each trial to remove odor of previous mice and the positions of the objects were similar during training and test. The time spent with novel objects was calculated as T_Novel_/(T_Novel_ + T_Familiar_) × 100. Specially, T_Novel_ refers to time spent with novel object and T_Familiar_ refers to time spent with familiar object. And rearing, NCD and locomotive activity were also evaluated (Matsumoto 2014; Hanania 2004) and involved in the statistical analysis as potential confounding factors.

### RNA isolation and real-time PCR

Samples of collected tissues and cultured cells were pulverized and then lysed using a QIAzol lysis buffer (Qiagen, Valencia, CA). Then, total RNA content was isolated from each sample by using a miRNeasy mini assay kit (Qiagen, Valencia, CA) according to manual instructions. Then, 2 μg of RNA in each sample were reversely transcribed into cDNA by using a Superscript II assay kit for reverse transcription (Invitrogen, Carlsbad, CA). Finally, real-time PCR was carried out by using a Prism 7900 HT real-time PCR system (Applied Biosystems, Foster City, CA) in conjunction with an SYBR green master mix (Applied Biosystems, Foster City, CA) following the instructions provided by the manufacturer. The relative expression of rno-miR-183-5p (Primer-F: 5′-TATGGCACTGGTAGAATTCAC-3′; Primer-R: 5′-GAACATGTCTGCGTATCTC-3′), rno-miR-34c-3p (Primer-F: 5′-AGGCAGTGTAGTTAGCTGATT-3′; Primer-F: 5′-GAACATGTCTGCGTATCTC-3′), rno-miR-210-3p (Primer-F: 5′-AGCCACTGCCCACAGCAC-3′; Primer-R: 5′-GAACATGTCTGCGTATCTC-3′), rno-miR-758-5p (Primer-F: 5′-TGGTTGACCAGAGAGCAC-3′; Primer-R: 5′-GAACATGTCTGCGTATCTC-3′), rno-miR-568 (Primer-F: 5′-ATGTATAAATGTATACACAC-3′; Primer-R: 5′-GAACATGTCTGCGTATCTC-3′), rno-miR-196c-3p (Primer-F: 5′-AGGTAGTTTCGTGTTGTTG-3′; Primer-R: 5′-GAACATGTCTGCGTATCTC-3′), Arc (Primer-F: 5′-TATTCAGGCTGGGTCCTGTC-3′; Primer-R: 5′-TGGAGCAGCTTATCCAGAGG-3′), Egr1 (Primer-F: 5′-AGCGAACAACCCTATGAGCA-3′; Primer-R: 5′-TCGTTTGCTGGGATAACTC-3′), Homer1 (Primer-F: 5′-GAAGTCGCAGGAGAAGATG-3′; Primer-R:5′-TGATTGCTGAATTGAATGTGTACC-3′), Narp (Primer-F: 5′-GTTCTGGGGAGTTCAAGGCA-3′; Primer-R: 5′-AGGAAGTGGCTCAGGCATCT-3′) and SIN3A mRNA (Primer-F: 5′-CAGAATGACACCAAGGTCCTGAG-3′; Primer-R: 5′-CATACGCAAGTGAGAGGTGTGG-3′) was quantified using the 2^−ΔΔ*CT*^ method.

### Cell culture and transfection

SH-SY5H cells were purchased from the American Type Culture Collection (ATCC, Manassas, VA) and maintained in Ham's F-12 medium added with 5% fetal bovine serum, 100 μg/ml streptomycin, as well as 100 U/ml penicillin. The cell culture was carried out in a 37 °C humidified incubator containing 95% air and 5% CO_2_. When the cells were 80% confluent, they were transfected with one of 6 candidate miRNAs, i.e., rno-miR-183-5p, rno-miR-34c-3p, rno-miR-210-3p, rno-miR-758-5p, rno-miR-568, or rno-miR-196c-3p, for 48 h before the mRNA and protein expression of SIN3A was detected. The transfection was carried out using Lipofectamine 2000 purchased from Invitrogen (Carlsbad, CA) following a routine transfection protocol.

### Vector construction, mutagenesis and luciferase assay

To clarify the regulatory relationship between SIN3A and rno-miR-183-5p, rno-miR-34c-3p, rno-miR-210-3p, rno-miR-758-5p, rno-miR-568 or rno-miR-196c-3p, the 3′ UTR sequences of SIN3A containing the binding sites for the above miRNAs were respectively cloned into different pcDNA luciferase vectors (Promega, Madison, WI) to produce vectors for wide type SIN3A 3′ UTRs. Then, site-directed mutagenesis was carried out using a Quick Change II site-directed mutagenesis assay kit (Stratagene, San Diego, CA) to generate mutant 3′ UTR sequences of SIN3A harboring the mutated binding sites for above miRNAs, respectively, and the mutant 3′ UTR sequences were also cloned into different pcDNA luciferase vectors to produce vectors for mutant SIN3A 3′ UTRs. In the next step, SH-SY5H cells were co-transfected with each of rno-miR-183-5p, rno-miR-34c-3p, rno-miR-210-3p, rno-miR-758-5p, rno-miR-568 or rno-miR-196c-3p in conjunction with wild-type or mutant 3′ UTR of SIN3A using Lipofectamine 2000. At 48 h post-transfection, the luciferase activity of transfected cells was assayed using a Bright Glo luciferase kit (Promega, Madison, WI) following the instructions provided by the manufacturer.

### Western blot analysis

Samples of collected tissues and cultured cells were prepared into a homogenate and centrifuged to collect the protein supernatant. Then, after quantifying protein concentrations by using a BCA assay kit (Pierce Biotechnology, Rockford, IL), an appropriate amount of sample protein was resolved by 10% denaturing SDS-PAGE and transferred onto PVDF membranes (Millipore, Bedford, MA), which were then blocked at room temperature for 1 h by using an Odyssey blocking reagent (Li-Cor, Lincoln, NE), incubated overnight in a 4 °C fridge with anti-SIN3A primary antibodies (ab129087, 1:1000 dilution, Abcam, Cambridge, MA), anti-Arc primary antibodies (ab18950, 1:1000 dilution, Abcam, Cambridge, MA), anti-Egr1 primary antibodies (ab133695, 1:1000 dilution, Abcam, Cambridge, MA), anti-Homer1 primary antibodies (ab184955, 1:1000 dilution, Abcam, Cambridge, MA) and anti-Narp primary antibodies (ab191563, 1:1000 dilution, Abcam, Cambridge, MA) and further incubated with HRP-conjugated secondary antibodies (ab6721, 1:2000 dilution, Abcam, Cambridge, MA). The relative protein expression was analyzed using an Odyssey imaging system (Li-Cor, Lincoln, NE).

### Immunohistochemistry assay

Samples of collected rat hippocampus tissues were sliced into 4 μm sections, fixed using PBS containing 4% paraformaldehyde, and quenched with 3% H_2_O_2_ to remove the activity of endogenous peroxidase. Then, after being blocked for 1 h in 5% BSA, the sections were treated overnight in a 4 °C fridge with anti-SIN3A primary antibodies (ab129087, 1:100 dilution, Abcam, Cambridge, MA). After washing and further incubation with biotin-labeled secondary antibodies (1:1000 dilution, Abcam, Cambridge, MA), the slides were incubated with horseradish peroxidase (HRP)-tagged avidin/streptavidin and counter-stained with 4′,6-diamidino-2-phenylindole (DAPI) before the positive expression of SIN3A was analyzed underneath a microscope.

### Statistical analysis

All experimental results were shown in mean ± SD. The comparison of different groups was carried out using Student’s *t*-test (for inter-group comparison) and one-way ANOVA (for multi-group comparison). A difference was deemed statistically significant if its probability was < 0.05, i.e., *p* < 0.05. All statistical analyses were done using SPSS 21.0 (IBM, Chicago, IL) and Prism 6.0 (GraphPad, San Diego, CA). Each experiment was repeated 3 times.

## Results

### Up-regulation of rno-miR-183-5p, rno-miR-34c-3p and rno-miR-210-3p in rats treated with EA

**Fig. 1:**
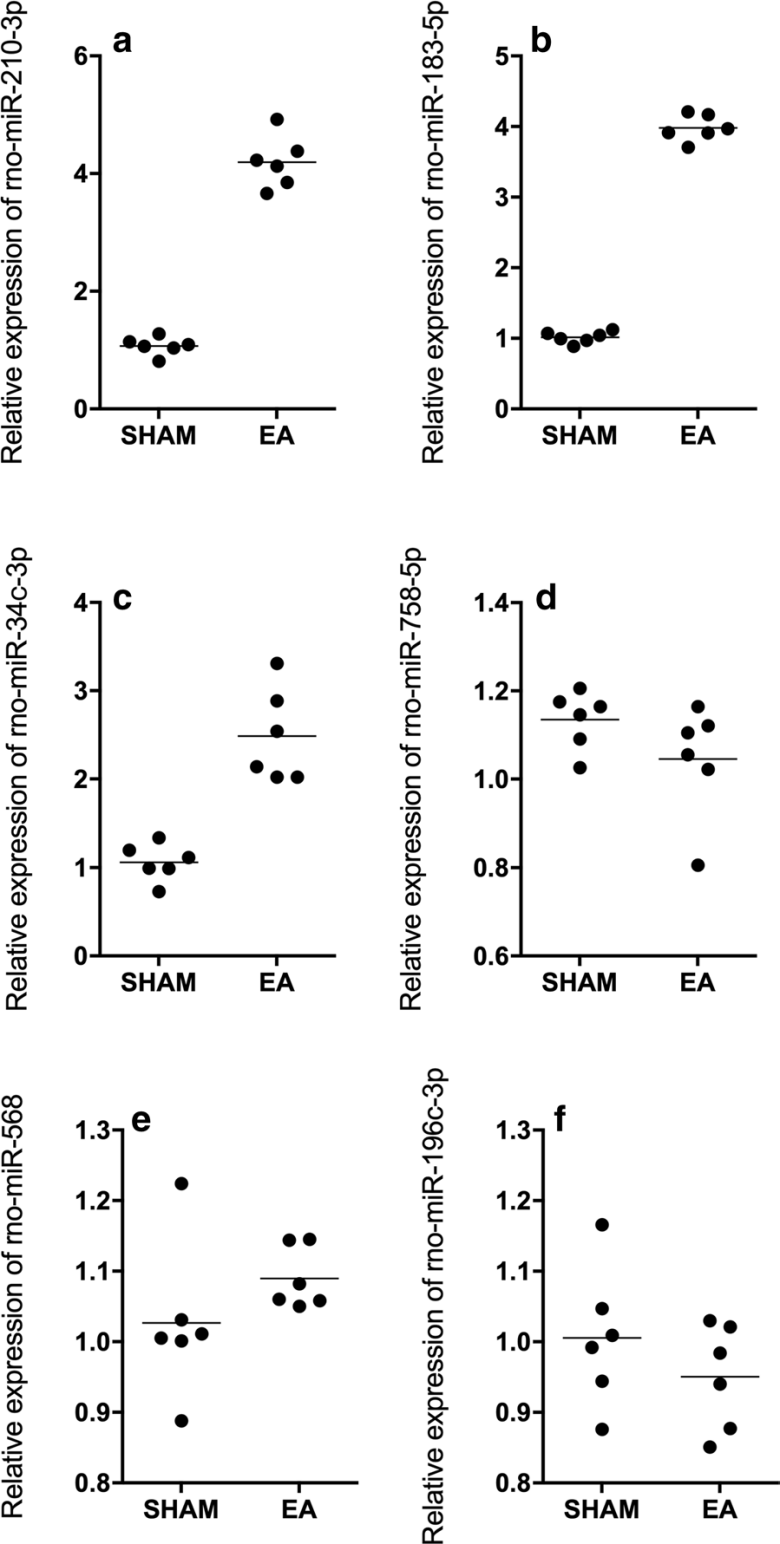
Rno-miR-183-5p, rno-miR-34c-3p and rno-miR-210-3p expression was enhanced in rats treated with EA (Real-time PCR; Technical repeats n = 3; Animal number in each group N = 6; *p value < 0.05, vs SHAM group). **a** Expression of rno-miR-183-5p was increased in EA-treated rats compared with sham-operated rats. **b** Expression of rno-miR-34c-3p was increased in EA-treated rats compared with sham-operated rats. **c** Expression of rno-miR-210-3p was increased in EA-treated rats compared with sham-operated rats. **d** Rno-miR-758-5p expression remained unchanged in sham rat group and EA rat group. **e** Rno-miR-568 expression remained unchanged in sham rat group and EA rat group. **f** Rno-miR-196c-3p expression remained unchanged in sham rat group and EA rat group

The expression of a group of miRNA candidates was evaluated in rats undergoing EA treatment. Among these miRNAs, the expression of rno-miR-183-5p (Fig. 1a), rno-miR-34c-3p (Fig. 1b) and rno-miR-210-3p (Fig. 1c) was significantly elevated in rats subjected to EA treatment. No obvious difference was observed for the expression of rno-miR-758-5p (Fig. 1d), rno-miR-568 (Fig. 1e) and rno-miR-196c-3p (Fig. 1f) between the two groups.

### Rno-miR-183-5p and rno-miR-210-3p inhibited the luciferase activity of wild-type SIN3A through binding to its 3′ UTR

**Fig. 2:**
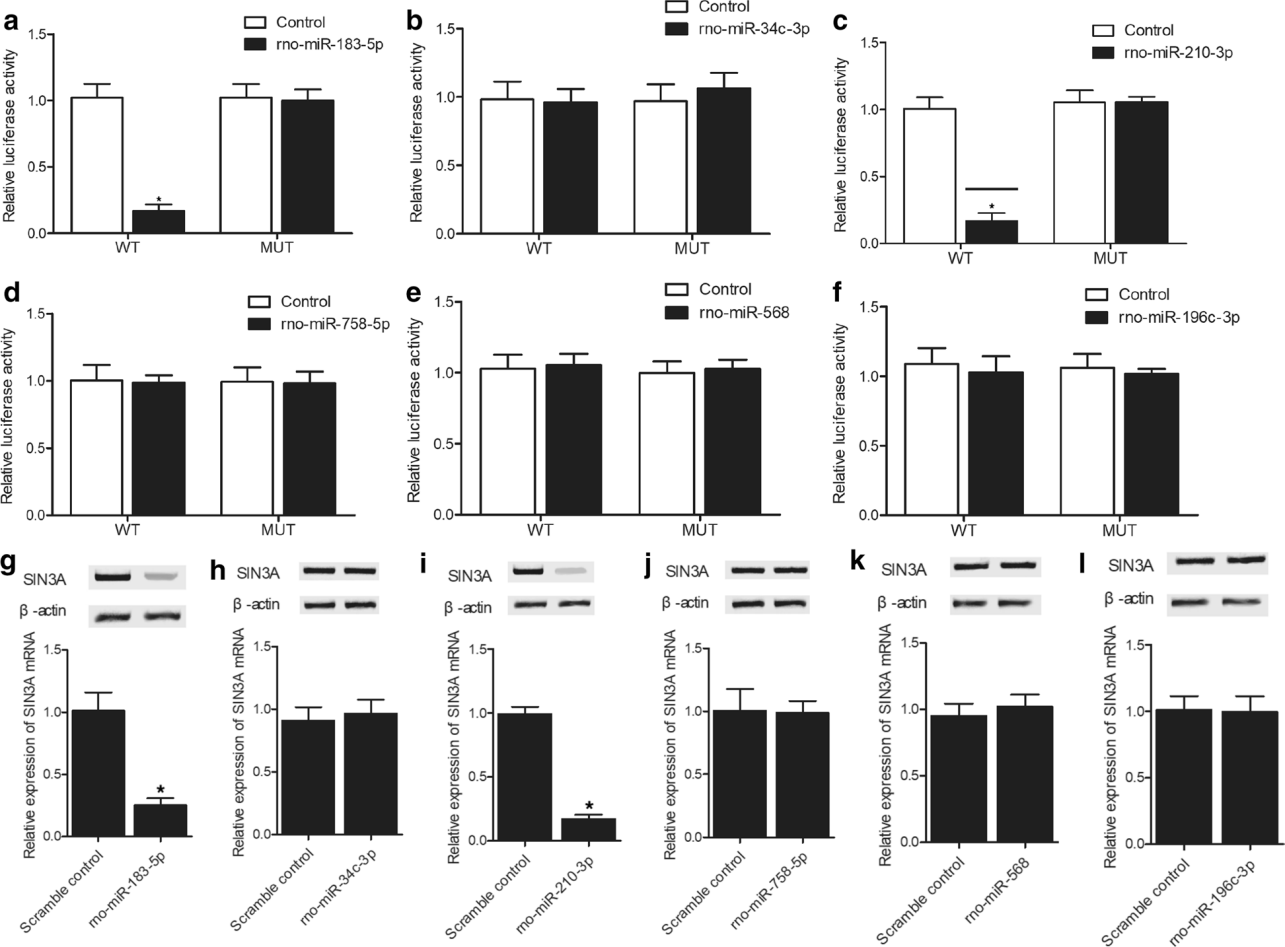
Rno-miR-183-5p and rno-miR-210-3p inhibited the luciferase activity of wild-type SIN3A through binding to its 3′ UTR (**a**–**f** Luciferase assay, N = 3, *p value < 0.05, vs control group). Rno-miR-183-5p and rno-miR-210-3p mimics suppressed the expression of SIN3A in SH-SY5H cells (**g**–**l** Real-time PCR and Western blot analysis; Technical repeats n = 3; *p value < 0.05, vs. scramble control group). **a** The luciferase activity of wild-type SIN3A was inhibited by rno-miR-183-5p. **b** Rno-miR-34c-3p showed no inhibitory effect on the luciferase activities of wild-type and mutant SIN3A. **c** The luciferase activity of wild-type SIN3A was inhibited by rno-miR-210-3p. **d** Rno-miR-758-5p showed no inhibitory effect on the luciferase activities of wild-type and mutant SIN3A. **e** Rno-miR-568 showed no inhibitory effect on the luciferase activities of wild-type and mutant SIN3A. **f** Rno-miR-196c-3p showed no inhibitory effect on the luciferase activities of wild-type and mutant SIN3A. **g** The expression of SIN3A was inhibited by rno-miR-183-5p in SH-SY5H cells. **h** Rno-miR-34c-3p showed no repressive effect on the SIN3A expression in SH-SY5H cells. **i** The expression of SIN3A was inhibited by rno-miR-210-3p in SH-SY5H cells. **j** Rno-miR-758-5p showed no repressive effect on the SIN3A expression in SH-SY5H cells. **k** Rno-miR-568 showed no repressive effect on the SIN3A expression in SH-SY5H cells. **l** Rno-miR-196c-3p showed no repressive effect on the SIN3A expression in SH-SY5H cells

SIN3A expression is closely correlated with SIA. To further explore the relationship between miRNAs and SIN3A, luciferase vectors containing wild-type and mutant SIN3A 3′ UTRs were established and co-transfected with candidate miRNAs to SH-SY5H cells. Rno-miR-183-5p (Fig. 2a) and rno-miR-210-3p (Fig. 2c) repressed the luciferase activity of wild-type SIN3A but had no inhibitory effect on mutant SIN3A. Rno-miR-34c-3p (Fig. 2b), rno-miR-758-5p (Fig. 2d), rno-miR-568 (Fig. 2e) and rno-miR-196c-3p (Fig. 2f) showed no effect on suppressing the luciferase activity of either wild-type or mutant SIN3A vectors. These results demonstrated that rno-miR-183-5p and rno-miR-210-3p were able to inhibit the expression of SIN3A through targeting the 3′ UTR of SIN3A.

### Rno-miR-183-5p and rno-miR-210-3p mimics suppressed the expression of SIN3A in SH-SY5H cells

**Fig. 3:**
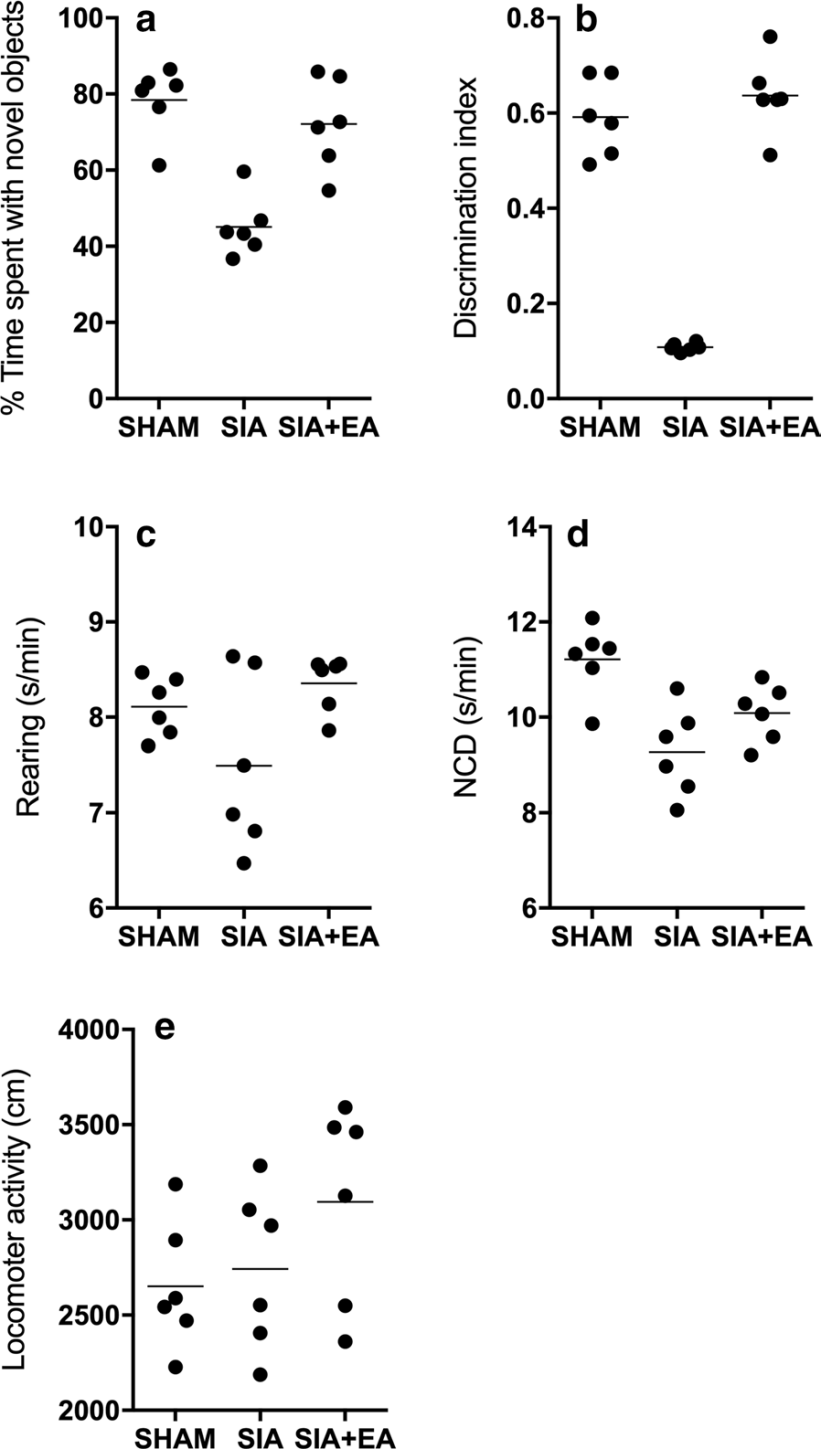
EA treatment improved memory consolidation in SIA rats (NOR test; Technical repeats n = 3; Animal number in each group N = 6; *p value < 0.05, vs sham group; **p value < 0.05, vs. SIA group). **a** Time spent with novel objects was reduced in SIA rats compared with the sham-operated rats, and the time spent with novel objects was increased by EA treatment in SIA + EA rats. **b** Discrimination index was evidently suppressed in SIA rats compared with sham-operated rats, while EA treatment restored the discrimination index in SIA rats. **c** No difference was spotted for the confounding parameter of rearing among all rat groups; **d** No difference was spotted for the confounding parameter of NCD among all rat groups; **e** No difference was spotted for the confounding parameter of locomotor activity among all rat groups

The mimics of rno-miR-183-5p and rno-miR-210-3p were transfected into SH-SY5H cells to evaluate their inhibitory effect on SIN3A expression. Based on the results obtained from the luciferase assays, rno-miR-183-5p (Fig. 2g) and rno-miR-210-3p (Fig. 2i) mimics apparently repressed the mRNA and protein expression of SIN3A in SH-SY5H cells. Rno-miR-34c-3p (Fig. 2h), rno-miR-758-5p (Fig. 3j), rno-miR-568(Fig. 2k) and rno-miR-196c-3p (Fig. 2l) showed no effect on SIN3A expression.

### EA treatment improved the memory consolidation of SIA rats

Scopolamine treatment was performed in rats to induce amnesia, followed by EA treatment. The NOR test was carried out to assess the discrimination index (DI) of SIA rats. The time spent with novel objects and the DI in SIA rats were dramatically decreased as compared with those in the SHAM control, while the treatment with EA effectively restored the time spent with novel objects (Fig. 3a) and the DI (Fig. 3b) of SIA rats closer to the normal levels. The confounding parameters including rearing (Fig. 3c), NCD (Fig. 3d) and locomotor activity (Fig. 3e) were similar among the rat groups.

### EA treatment restored the dysregulated expression of rno-miR-183-5p, rno-miR-210-3p and SIN3A in SIA rats

**Fig. 4:**
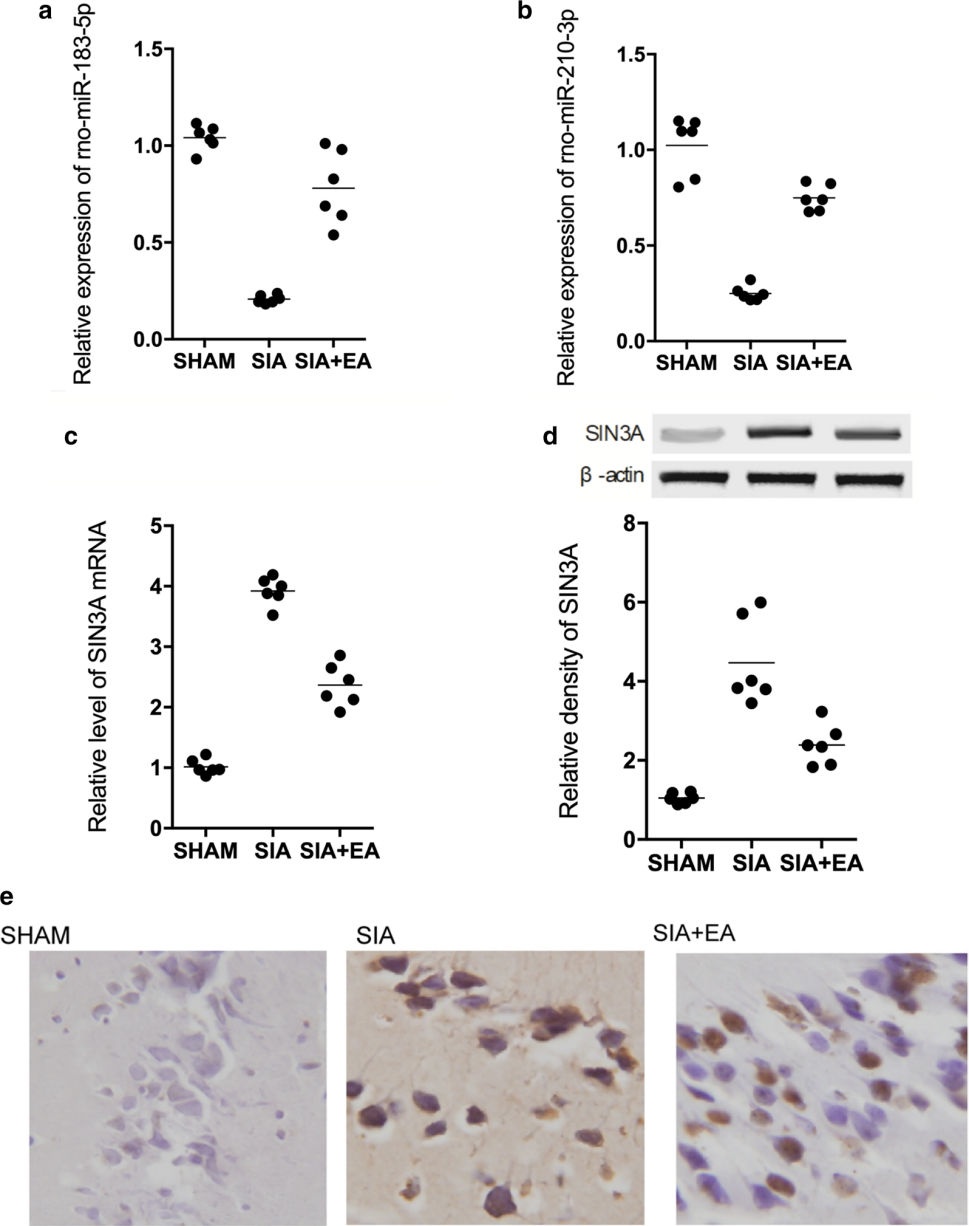
EA treatment restored up-regulated rno-miR-183-5p and rno-miR-210-3p, and down-regulated SIN3A mRNA and protein in SIA rats (**a**–**c** Real-time PCR; **d** Western blot; **e** IHC analysis; Technical repeats n = 3; Animal number in each group N = 6; *p value < 0.05, vs sham group; **p value < 0.05, vs. SIA group). **a** Rno-miR-183-5p expression was the lowest in SIA rats, while EA treatment partly restored the rno-miR-183-5p expression in SIA rats. **b** Rno-miR-210-3p expression was the lowest in SIA rats, while EA treatment partly restored the rno-miR-210-3p expression in SIA rats. **c** The level of SIN3A mRNA was the highest in SIA rats, while EA treatment partly restored the SIN3A mRNA expression in SIA rats. **d** The level of SIN3A protein was the highest in SIA rats, while EA treatment partly restored the SIN3A protein level in SIA rats. **e** EA treatment reduced SIN3A expression in the hippocampus of SIA rats

In rats suffering from scopolamine-induced amnesia, the expression of rno-miR-183-5p and rno-miR-210-3p was notably decreased, while the EA treatment remarkably recovered the down-regulated expression of rno-miR-183-5p (Fig. 4a) and rno-miR-210-3p (Fig. 4b) in SIA rats. However, the mRNA and protein expression of SIN3A was obviously elevated in SIA rats, while the EA treatment remarkably attenuated the abnormal mRNA (Fig. 4c) and protein (Fig. 4d) levels of SIN3A. Also, since amnesia is closely related to the hippocampus of the brain, we further performed IHC on the hippocampus tissues of rats to analyze their expression of SIN3A (Fig. 4e). Accordingly, SIN3A expression was apparently increased in SIA rats, while the treatment with EA down-regulated the expression of SIN3A in the hippocampus of SIA rats.

### EA treatment promoted the inhibited expression of neuronal IEGs including Arc, Egr1, Homer1 and Narp in the hippocampus of SIA rats

**Fig. 5:**
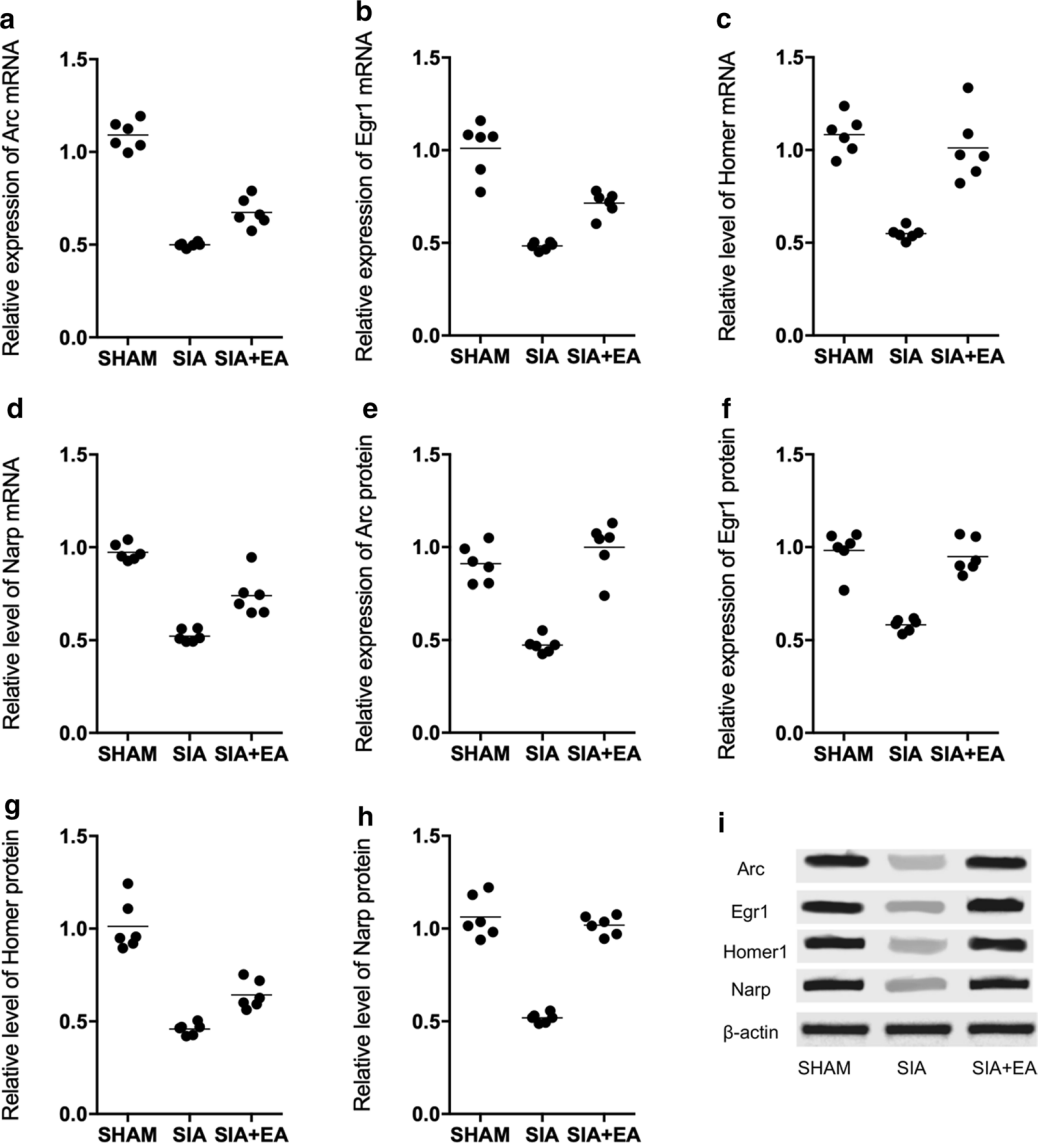
The inhibited mRNA and protein expression of Arc, Egr1, Homer1 and Narp in the hippocampus of SIA rats was up-regulated by EA treatment (**a** Real-time PCR; **b** Western blot; Technical repeats n = 3; Animal number in each group N = 6; *p value < 0.05, vs. sham group; **p value < 0.05, vs. SIA group). **a** Compared with the sham-operated rats, the gene expression of Arc mRNA was decreased in the hippocampus of SIA rats, and EA treatment partly restored the decreased expression of above genes. **b** Compared with the sham-operated rats, the gene expression of Egr1 mRNA was decreased in the hippocampus of SIA rats, and EA treatment partly restored the decreased expression of above genes. **c** Compared with the sham-operated rats, the gene expression of Homer1 mRNA was decreased in the hippocampus of SIA rats, and EA treatment partly restored the decreased expression of above genes. **d** Compared with the sham-operated rats, the gene expression of Narp mRNA was decreased in the hippocampus of SIA rats, and EA treatment partly restored the decreased expression of above genes. **e** Compared with the sham-operated rats, the protein expression of Arc was decreased in the hippocampus of SIA rats, and EA treatment partly restored the decreased expression of above proteins. **f** Compared with the sham-operated rats, the protein expression of Egr1 was decreased in the hippocampus of SIA rats, and EA treatment partly restored the decreased expression of above proteins. **g** Compared with the sham-operated rats, the protein expression of Homer1 was decreased in the hippocampus of SIA rats, and EA treatment partly restored the decreased expression of above proteins. **h** Compared with the sham-operated rats, the protein expression of Narp was decreased in the hippocampus of SIA rats, and EA treatment partly restored the decreased expression of above proteins. **i** Western blot of the protein expression of Arc, Egr1, Homer1 and Narp of all rat groups

By performing qPCR on the expression of neuronal IEGs including Arc, Egr1, Homer1 and Narp, it was demonstrated that in the hippocampus of SIA rats, the gene expression of Arc (Fig. 5a), Egr1 (Fig. 5b), Homer1 (Fig. 5c) and Narp (Fig. 5d) was all evidently down-regulated, while the EA treatment alleviated the down-regulation of these genes. Moreover, the Western blot analysis also showed a similar trend in the protein expression of Arc, Egr1, Homer1 and Narp in different rat groups (Fig. 5e–i).

## Discussion

The dosing of scopolamine can induce the impairment of cognitive functions in experimental animals by changing the levels of oxidative stress in the brain of rats (Fan et al. 2005). In addition, several compounds derived from plants show a promising efficacy in augmenting cognitive behaviors by blocking the functions of scopolamine. As a result, the above-mentioned compounds have been tested in animal models to treat memory loss (Sawmiller et al. 2016). Interestingly, EA therapies can rescue long-term impairments of memory functions in rats suffering from dementia by protecting the integrity of neuron cells while reducing the expression of Noxa, Bax, and P53 in rat hippocampus (Zhu and Zeng 2011; Wang et al. 2004). These results suggested that several signaling pathways may be involved in the role of EA therapies in the treatment of vascular dementia (Duan et al. 2016). In this study, we compared the expression of 6 candidate miRNAs in rats to evaluate the effect of EA on their expression. We found that rno-miR-183-5p, rno-miR-34c-3p and rno-miR-210-3p in rats treated with EA were significantly up-regulated. In addition, we performed the NOR test on rats suffering from SIA. We found that EA treatment improved the level of memory consolidation in SIA rats. Furthermore, we analyzed the expression of rno-miR-183-5p, rno-miR-210-3p and SIN3A in SIA rats, and showed that the EA treatment effectively prevented the dysregulated expression of rno-miR-183-5p, rno-miR-210-3p and SIN3A in SIA rats. Growing evidence suggests that the treatment by EA can alleviate neuronal disorders caused by neuropathy (Bao et al. (2017; Duran-Aniotz and Hetz 2016). Jing et al. demonstrated that the treatment by EA can apparently enhance memory as well as learning capacities. In two recent publications, it was demonstrated that the treatment by EA can apparently enhance the ability of long term memory and learning in mice suffering from the AD disease (Dubois et al. 2007; Wang et al. 2016; Gardener et al. 2016). And the treatment by EA was demonstrated to reduce the time required to complete face recognition while decreasing the levels of psychological stress (Laforce et al. 2014).

A miRNA, miR-210, may regulate the process of cell apoptosis as well as cell proliferation by directly reducing the expression level of SIN3A. In addition, the regulatory effect of miR-210 inhibitors on the process of cell apoptosis as well as cell proliferation could be blocked by silencing the mRNA expression of SIN3A, suggesting that miR-210 exerts its effects by regulating the expression of SIN3A. In addition, a cluster of miRNAs containing miR-183, miR-96, and miR-182 can enhance the ability of brain learning by regulating the expression levels of a memory suppressor, i.e., protein phosphatase 1 (PP1) (Woldemichael et al. 2016). While the biogenesis process of multiple miRNAs highly expressed in neuron cells can be regulated by miR-183, miR-96, and miR-182, these miRNAs mainly exert their effects by elevating the expression levels of PP1 in the nuclear domain of cells (Jawaid et al. 2019). In this study, we transfected SH-SY5H cells with miRNA mimics to evaluate their effect on SIN3A expression. Rno-miR-183-5p and rno-miR-210-3p mimics suppressed the expression of SIN3A in SH-SY5H cells. Moreover, we performed luciferase assay to explore the inhibitory effect of candidate miRNAs on SIN3A. Rno-miR-183-5p and rno-miR-210-3p inhibited the luciferase activity of SIN3A through binding to its 3′ UTR.

As a large-sized scaffold protein, SIN3A contains several domains of the paired amphipathic helix as well as an HDAC interaction domain. The paired amphipathic helix domains in SIN3A are responsible for recognizing as well as binding to certain transcriptional factors including MAD and MAX. On the other hand, the HDAC interaction domain can dock HDAC1 proteins as well as HDAC2 proteins to SIN3A, so as to repress the expression of target genes of SIN3A (Kadamb et al. 2013). In addition, the treatment using scopolamine can decrease the levels of acetylation in the promoters of H3K9 as well as H3K14 genes, while the silencing of SIN3A expression elevated the levels of acetylation in the promoters of H3K9 as well as H3K14 genes (Peleg et al. 2010). In addition, the silencing of SIN3A expression can also alter the levels of acetylation in the promoter of the H4K12 gene to deregulate the expression of multiple genes involved in memory consolidation. Similarly, it was reported by Fischer et al. that the induced acetylation of the H3K9 gene by environmental factors can restore the consolidation of spatial memory (Fischer et al. 2007).

Moreover, Srivas et al. also reported that in the hippocampus of rats with amnesia, the dysregulated expression of SIN3A is correlated with the expression of neuronal IEGs including Arc, Egr1, Homer1 and Narp (Srivas and Thakur 2018). And IEGs are defined as a family of synaptic plasticity genes that are down-regulated during amnesia, aging and several neurodegenerative disorders (Davis et al. 2003; Penner et al. 2011; Srivas and Thakur 2017; Singh and Thakur 2014). They encode both regulatory transcription factors and effector proteins to actively participate in the process of synaptic plasticity, glutamate receptor clustering at the synapse, and memory consolidation (Srivas and Thakur 2018; Loebrich and Nedivi 2009; Abel and Lattal 2001b). Therefore, the promoted gene and protein expression of Arc, Egr1, Homer1 and Narp by EA treatment also indicated the therapeutic effect of EA on scopolamine-induced amnesia.

However, there are limitations of our study. As an important part of Chinese Traditional Medicine, EA could be categorized as experience-based medicine and initially developed in clinical practice. The therapeutic effect of EA treatment in this study was only evaluate in animal models, and further validation on human subjects should be performed. In addition, the involved signaling pathways and genes investigated in this study was limited and there must be other pathways and genes besides those observed in this study involved in the molecular mechanism underlying the therapeutic effect of EA.

## Conclusion

In summary, the findings of this study demonstrated that scopolamine-induced amnesia was associated with downregulated expression of miR-210/miR-183 and upregulated expression of SIN3A. Furthermore, treatment with EA alleviated scopolamine-induced amnesia in rats and was associated with upregulated expression of miR-210/miR-183 and downregulated expression of SIN3A.

## Data Availability

All relevant data are in the paper.
